# Association between E469K polymorphism in the ICAM1 gene and the risk of diabetic nephropathy: a meta-analysis

**DOI:** 10.1186/s12944-018-0922-2

**Published:** 2018-12-26

**Authors:** Liya Liu, Dongling He, Ling Fang, Xiaojie Yan

**Affiliations:** 1 Department of Pharmacy, Shenzhen Hospital, Southern Medical University, Shenzhen, 518000 Guangdong China; 2 Department of Nephrology, The Eight Affiliated Hospital, SUNYAT-SEN University, Shenzhen, Guangzhou, 518033 China; 3 Department of Endocrinology, Shenzhen Hospital, Southern Medical University, No. 1333, New Lake Road, Baoan District, Shenzhen, 518000 Guangdong China

**Keywords:** Intercellular adhesion molecule 1 (ICAM1), Diabetic nephropathy (DN), Diabetes mellitus (DM), E469K

## Abstract

**Background:**

Inflammation may be a key pathophysiological mechanism in diabetic nephropathy (DN). Intercellular adhesion molecule 1 (ICAM1) is an acute phase marker of inflammation. ICAM1 rs5498 has been reported to be associated with the risk of DN. However, the previous findings were conflicting due to the limited sample sizes, different methodologies and ethnicities. Therefore, this study aimed to investigate the genetic association between ICAM1 rs5498 and the risk of DN.

**Methods:**

Two investigators independently searched the studies from the databases PubMed, Web of Science, the Cochrane Library, Chinese National Knowledge Infrastructure (CNKI) and Embase. Pooled odds ratios (ORs) with 95% confidence intervals (CIs) were used to assess the associations.

**Results:**

No significant association was detected between ICAM1 rs5498 and DN susceptibility in allelic and recessive models (*p* > 0.05). However, significant reduction of frequencies of the dominant model of ICAM1 rs5498 was only detected in the Caucasian subgroup (OR = 0.80; 95% CI = [0.65, 0.99], *p* = 0.04) and type 1 diabetes mellitus subgroup (OR = 0.80; 95% CI = [0.65, 0.99], p = 0.04).

**Conclusions:**

Thus, ICAM1 rs5498 might be a risk factor for DN in Caucasians and type 1 diabetes mellitus patients, which suggested that ICAM1 rs5498 might help in early diagnosis and prevention of this disease. Further studies were needed to clarify the biochemical function and pathological role of ICAM1 rs5498 in the risk of DN.

**Electronic supplementary material:**

The online version of this article (10.1186/s12944-018-0922-2) contains supplementary material, which is available to authorized users.

## Introduction

Diabetes mellitus (DM) is a metabolic disorder associated with chronic micro and macro vascular complications [1, 2]. One of the worst chronic microvascular complication is diabetic nephropathy (DN) [3]. Approximately 40–45% of patients with type 1 DM (T1DM) and 30% with type 2 DM (T2DM) develop DN [4, 5]. DN is the most common single cause of end-stage renal disease (ESRD) [6]. The patients with DN exhibit persistent proteinuria, hypertension, declining renal function, and increased premature mortality, primarily as a result of cardiovascular disease [7]. Multi-factorial diseases including genetic and environmental factors are known to influence DM and DN [8, 9].

Genome-wide scans have predicted that genes located at chromosome 19p13 might be susceptible to T1DM [10]. The intercellular adhesion molecule 1 (ICAM1) gene is located in 19p13 and encodes a 90-kD cell surface glycoprotein of the Ig super family involved in the firm attachment of leukocytes to endothelium [11]. Both mRNA and protein levels of ICAM1 were significantly increased in animal models of DN with T1DM and T2DM [12, 13]. Therefore, the ICAM1 gene is a strong positional and biological candidate for the susceptibility to the development of T1DM, T2DM, and DN.

Genetic association studies involving a common non-synonymous single nucleotide polymorphism (SNP) of ICAM1 (rs5498 E469K) in T1DM patients have been reported [14, 15]. Guja et al. first demonstrated that transmission of the G allele of SNP K469E (A/G) was increased in Romanian T1DM families [16]. Furthermore, a case-control study conducted by Ma et al. [17, 18] reported that the rs5498 E469K (A/G) in the ICAM1 gene might be associated with T1DM-DN in GoKinD and Swedish Caucasians. Besides, Chen et al. revealed that the 469G allele might be a susceptibility factor for T2DM with DN in Zhuang populations in the northern regions of China [19]. However, Wang et al. found that the frequency of AA genotype of E469K was significantly increased in T2DM Chinese with DN [20]. Ren et al. also reported that rs5498A allele of SNP rs5498 was associated with T2DM-DN in Chinese [21].

Due to limited sample sizes and inconsistent results of individual studies, this meta-analysis aimed to systematically evaluate the genetic association between rs5498 E469K and DN susceptibility.

## Methods

### Literature search strategy

This meta-analysis followed the Cochrane Collaboration definition and PRISMA 2009 guidelines for meta-analysis and systematic review [22]. To identify eligible studies, we systematically searched PubMed, Embase, the Cochrane Library and Chinese National Knowledge Infrastructure (CNKI) databases. The keywords used for search were as follows: “intercellular adhesion molecule-1 or ICAM-1” and “polymorphism OR variant” and “diabetic nephropathy or DN”. No limitations on language and publication year was detected. The last search was updated on October 15, 2017. Furthermore, references of all relevant studies were retrieved to identify additional eligible studies.

### Inclusion/exclusion criteria

Inclusion criteria: (1) case-control studies; (2) evaluating the association between ICAM1 rs5498 polymorphism and DN risk; (3) available genotype frequencies; (4) the genotype distribution in control groups was in the Hardy-Weinberg equilibrium (HWE). Exclusion criteria: (1) replicated studies; (2) studies in which the genotype or allele frequency could not be obtained; (3) data from meetings, case-reports, reviews or abstracts; (4) control group did not conform to HWE.

### Data extraction

Two researchers (Liya Liu and Dongling He) independently extracted the data from each eligible publication, and discussed to reach a consensus in case of disagreements. For each study, the following data were reviewed and collected: First author, year of publication, ethnicity, number of cases and controls, type of DM, mean age, gender, duration time, HbA1c, Body Mass Index (BMI), creatinine, High Density Lipoprotein (HDL), Systolic blood pressure (SBP), and Diastolic blood pressure (DBP).

### Quality assessment

The study quality was independently assessed by Xiaojie Yan and Ling Fang. Any discrepancies in the assessment were resolved by the third author Xiaojie Yan. The quality of each included study was assessed according to the Newcastle–Ottawa Scale (NOS) (Additional file 1: Table S1) [23]. Group selection, comparability and assessment of outcome or exposure were taken into account and given a corresponding score. Total score ranged from 0 (lowest quality) to 9 (highest quality). A study with a score of 6 or higher was selected in present meta-analysis.

### Statistical analysis

Statistical analysis was conducted using the Stata software (version 12.0; Stata Corp LP, College Station, TX, USA) and RevMan software (version 5.1). The pooled ORs were performed for null versus present genotype. The statistical significance of the pooled ORs under different genetic models (allelic, recessive, additive and dominant) were determined by Z-test, and considered significant when *P* < 0.05. Heterogeneity assumption was tested by the chi-square-based Q-test. An I^2^ value of < 50% for the Q-test indicated a lack of heterogeneity among studies, so the pooled OR estimate of each study was calculated by the fixed-effects model (the Mantel-Haenszel method). Otherwise, the random-effects model (the DerSimonian and Laird method) was used. Sensitivity analysis was performed to assess the effects of every individual study on the pooled results and the stability of results. Publication bias was assessed with funnel plots by Begg’s and Egger’s tests.

## Results

### Study characteristics

**Fig. 1:**
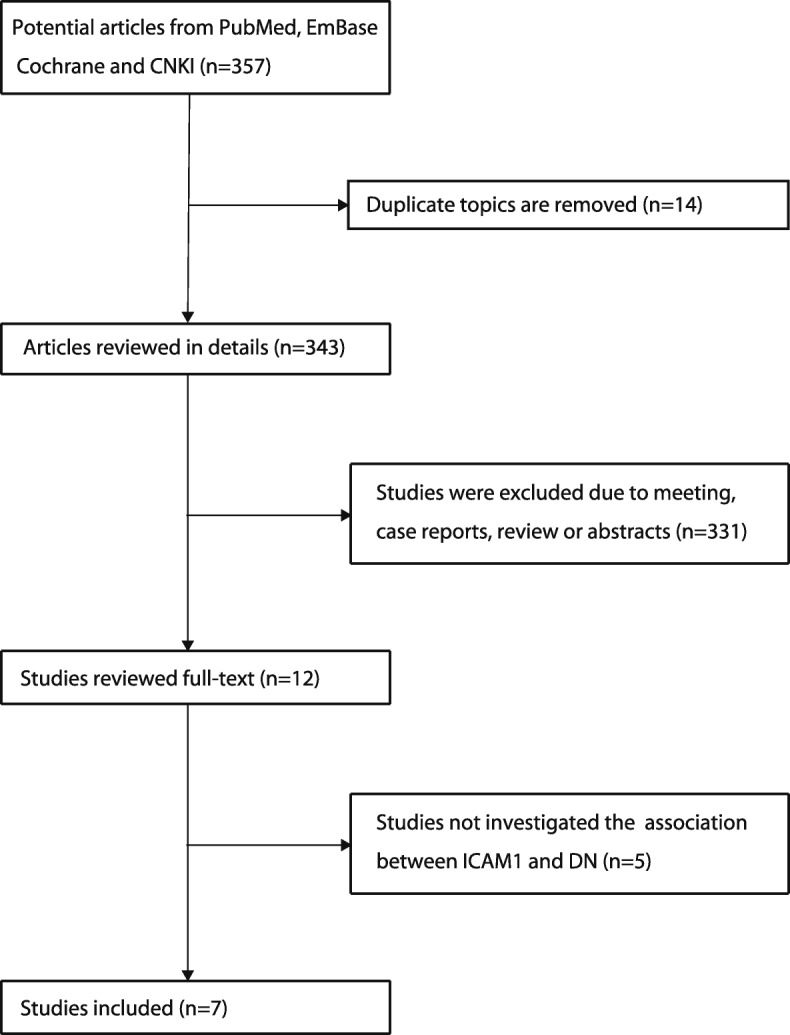
PRISMA flow chart of inclusion and exclusion criteria of studies

A total of 357 studies were retrieved through the literature search. A total of 14 duplicate reports were excluded. After screening the titles, abstracts, and full-texts, seven eligible studies were included in the present meta-analysis and used for data extraction [17–21, 24, 25]. A flowchart of the study selection process is shown in Fig. 1. The data of the selected studies is presented in Table 1.

**Table 1:** Main characteristics of the studies for polymorphisms included in the meta-analysis

First author	Year	Ethnicity	Case/ Control	DM	Mean age	Gender (M:F)	Duration (years)	Creatinine (μmol/l)	Urea nitrogen (mmol/l)	Plasma glucose (mmol/l)	HbA1c (mmol/mol)	HDL (mg/dL)	LDL (mg/dL)	Uaer (mg /24 h)	Quality
Ma	2008	GoKinD	662/620	T1D	44 ± 7/ 40 ± 8	350/312: 251/369	31 ± 8/ 26 ± 8	37.45 ± 33.51/ 15.39 ± 2.74	NA	NA	NA	53.6 ± 17.5/ 59.0 ± 15.8	NA	NA	9
Ma	2006	Swedish	196/236	T1D	46 ± 12/ 44 ± 12	103/93: 105/131	34 ± 12/ 29 ± 10	NA	NA	NA	7.4 ± 1.35/ 7.0 ± 1.15	NA	NA	NA	9
Seman	2015	Malaysia	303/277	T2D	57 ± 10/ 56 ± 10	166/137: 120/157	13 ± 8/ 13 ± 8	202.3 ± 171.2/ 90.8 ± 71.1	NA	6.4 ± 0.7/ 5.3 ± 2.1	8.8 ± 2.2/ 8.1 ± 1.9	NA	NA	NA	6
Ren	2015	Chinese	402/385	T2D	63.8 ± 11.4/ 66.4 ± 11.2	204/198: 191/194	14.3 ± 6.8/ 15.6 ± 5.9	69.8 ± 43.6/ 76.6 ± 55.9	6.1 ± 2.5/ 6.2 ± 2.7	8.9 ± 3.6 /7.5 ± 2.7	68.4 ± 22.1/ 64.0 ± 18.0	1.3 ± 0.5/ 1.3 ± 0.8	2.6 ± 0.8/ 2.5 ± 0.8	NA	8
Chen	2010	Chinese	94/95	T2D	57.69 ± 9.72/ 55.72 ± 9.98	42/52: 44/51	85.85 ± 79.76/ 83.15 ± 50.85	NA	NA	NA	NA	NA	NA	NA	8
wang	2006	Chinese	110/100	T2D	61.7 ± 7.1/ 60.2 ± 6.9	56/54: 50/50	10.2 ± 5.9/ 9.7 ± 6.1	NA	NA	NA	NA	0.92 ± 0.29/ 1.11 ± 0.21	3.61 ± 0.60/ 3.10 ± 0.67	1390(990–2560)/< 30	7
Oguz	2015	Turkish	138/138	NC	54.77 ± 2.49/ 50.1 ± 8	46/92: 49/89	na	NA	NA	170 ± 21.2/89 ± 8.7	11.36 ± 1.8/ 5.5 ± 0.4	39.18 ± 13.8/ 51.65 ± 4.2	130.00 ± 20/ 96.60 ± 22.77	NA	6

Abbreviations: *DM* Diabetes mellitus, *T1D* type 1 Diabetes, *T2D* type 2 Diabetes, *M* Male, *F* Female, *HDL* High Density Lipoprotein, *LDL* Low Density Lipoprotein, *NA* not avaiable

### Meta-analysis results

**Fig. 2:**
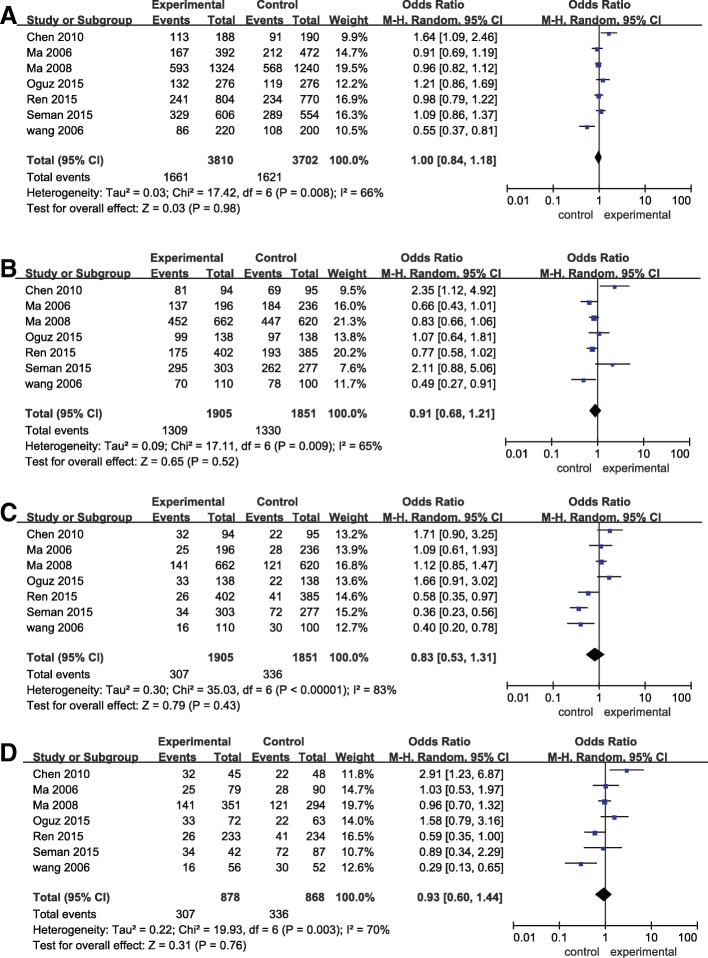
Forest plots of odds ratios for the association between ICAM1 rs5498 and diabetic nephropathy. (**a**): allelic model; (**b**) dominant model; (**c**) recessive model; (**d**) additive model

After all the extracted data were pooled, a total of 1901 cases and 1847 controls were available for ICAM1 analysis. No significant relationship between the genetic models (allelic, dominant, additive and recessive) of ICAM1 and increased risk of DN was detected (*p* > 0.05) (Fig. 2). Significant heterogeneity was observed in the total group in allelic, dominant, additive and recessive models of ICAM1 (I^2^ > 50%) (Fig. 2). Thus, subgroup analyses based on ethnicity, sample size, and type of DM were conducted. In the subgroup analyses for different countries group, the dominant model of ICAM1 showed decreased risk of DN among Caucasians (OR = 0.80; 95% CI = [0.65, 0.99]). Also, analyses stratified by different types of DM showed a significant association of reduced risk of dominant model of ICAM1 in T1DM subgroup (OR = 0.80; 95% CI = [0.65, 0.99], *p* = 0.04), but not in T2DM subgroup (OR = 1.16; 95% CI = [0.63, 2.14], *p* = 0.04). However, analyses stratified by sample size (sample size > 1000 and sample size < 1000) showed no significant association between allelic, dominant, additive and recessive models of ICAM1 and the risk of DN (*p* > 0.05) (Table 2).

**Table 2:** The results of meta-analysis for ICAM1 rs5498 and diabetic nephropathy

SNPs (minor allele)	Genetic Model	subgroup	Number of studies	Numbers		Test of association		Model	Test of heterogeneity	
				case	control	OR[95% CI]	*p*-Value		*P* value	I^2^ (%)
rs5498	Allelic	total	7	3802	3694	0.93 [0.77, 1.11]	0.41	R	0.003	69
		Asian	5	2086	1986	0.93 [0.69, 1.25]	0.64	R	0.0008	79
		Caucasian	2	1716	1708	0.94 [0.82, 1.08]	0.40	F	0.67	0
		> 1000	1	1324	1240	0.96 [0.82, 1.12]	0.60	NA	NA	NA
		< 1000	6	2478	2454	0.92 [0.73, 1.17]	0.50	R	0.002	74
		T1D-DN	2	1716	1708	0.94 [0.82, 1.08]	0.40	F	0.67	0
		T2D-DN	4	1810	1710	0.87 [0.63, 1.22]	0.43	R	0.001	81
	Dominant	total	7	1901	1847	0.96 [0.73, 1.27]	0.76	R	0.01	62
		Asian	5	1043	993	1.12 [0.71, 1.76]	0.63	R	0.01	70
		Caucasian	2	858	854	0.80 [0.65, 0.99]	0.04	F	0.55	0
		> 1000	1	662	620	0.83 [0.66, 1.06]	0.14	NA	NA	NA
		< 1000	6	1239	1227	1.02 [0.70, 1.48]	0.93	R	0.01	67
		T1D-DN	2	858	854	0.80 [0.65, 0.99]	0.04	F	0.55	0
		T2D-DN	4	905	855	1.16 [0.63, 2.14]	0.64	R	0.004	77
	Recessive	total	7	1901	1847	0.83 [0.53, 1.31]	0.43	R	< 0.00001	83
		Asian	5	1043	993	0.74 [0.38, 1.43]	0.37	R	< 0.0001	85
		Caucasian	2	858	854	1.11 [0.87, 1.42]	0.41	F	0.91	0
		> 1000	1	662	620	1.12 [0.85, 1.47]	0.43	NA	NA	NA
		< 1000	6	1239	1227	0.79 [0.45, 1.38]	0.40	R	< 0.0001	83
		T1D-DN	2	858	854	1.11 [0.87, 1.42]	0.41	F	0.91	0
		T2D-DN	4	905	855	0.61 [0.31, 1.17]	0.13	R	0.0009	82
	Additive	total	7	878	868	0.93 [0.60, 1.44]	0.76	R	0.003	70
		Asian	5	448	484	0.92 [0.44, 1.92]	0.82	R	0.0006	80
		Caucasian	2	166	149	0.97 [0.73, 1.29]	0.84	F	0.86	0
		> 1000	1	141	121	0.96 [0.70, 1.32]	0.80	NA	NA	NA
		< 1000	6	527	574	0.93 [0.52, 1.68]	0.82	R	0.001	75
		T1D-DN	2	166	149	0.97 [0.73, 1.29]	0.84	F	0.86	0
		T2D-DN	4	376	421	0.80 [0.33, 1.92]	0.62	R	0.001	81

Abbreviations: *SNP* single nucleotide polymorphism, *T1D* type 1 Diabetes, *T2D* type 2 Diabetes, *DN* diabetic nephropathy, *ICAM1* intercellular adhesion molecule-1;

*F* fixed model, *R* random model, *OR* odds ratio, *CI* confidence interval, *NA*: not available

### Sensitivity analysis

**Fig. 3:**
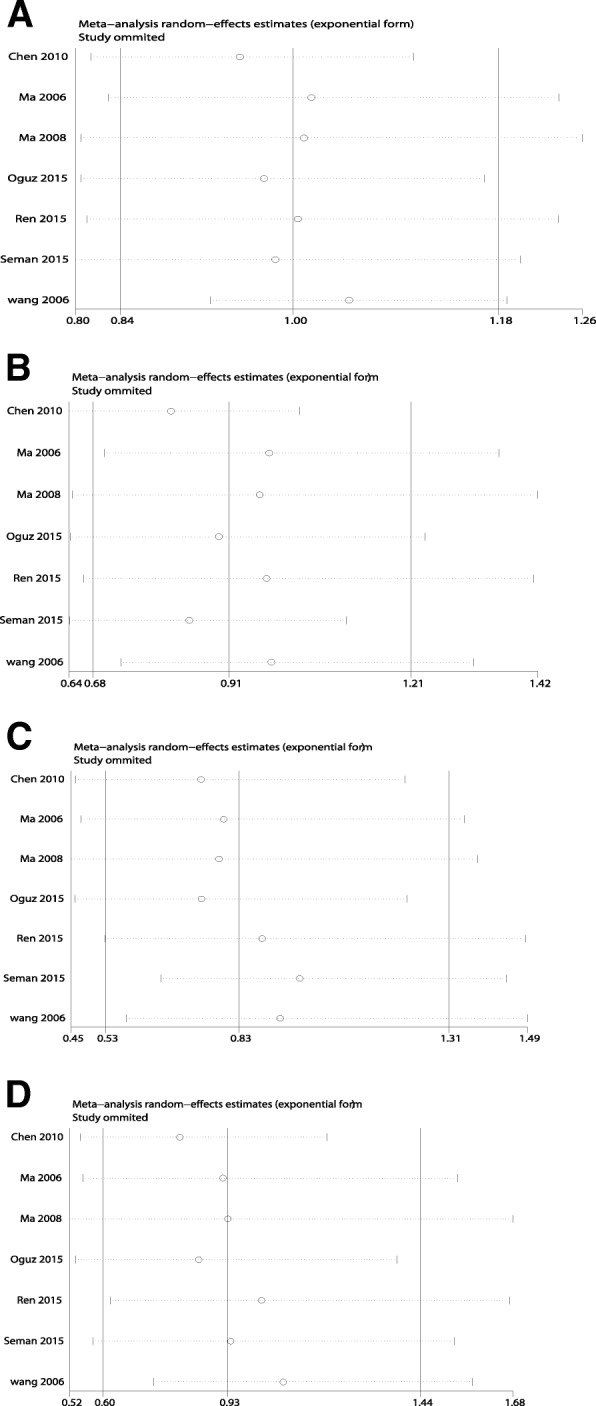
The influence of each study after removal of individual studies for ICAM1 rs5498. (**a**): allelic model; (**b**) dominant model; (**c**) recessive model; (**d**) additive model

Sensitivity analysis was performed to determine the influence of a single study on the overall risk estimate by individually excluding each study. The ORs were not significantly altered in each adverse complication (Fig. 3).

### Publication bias

**Fig. 4:**
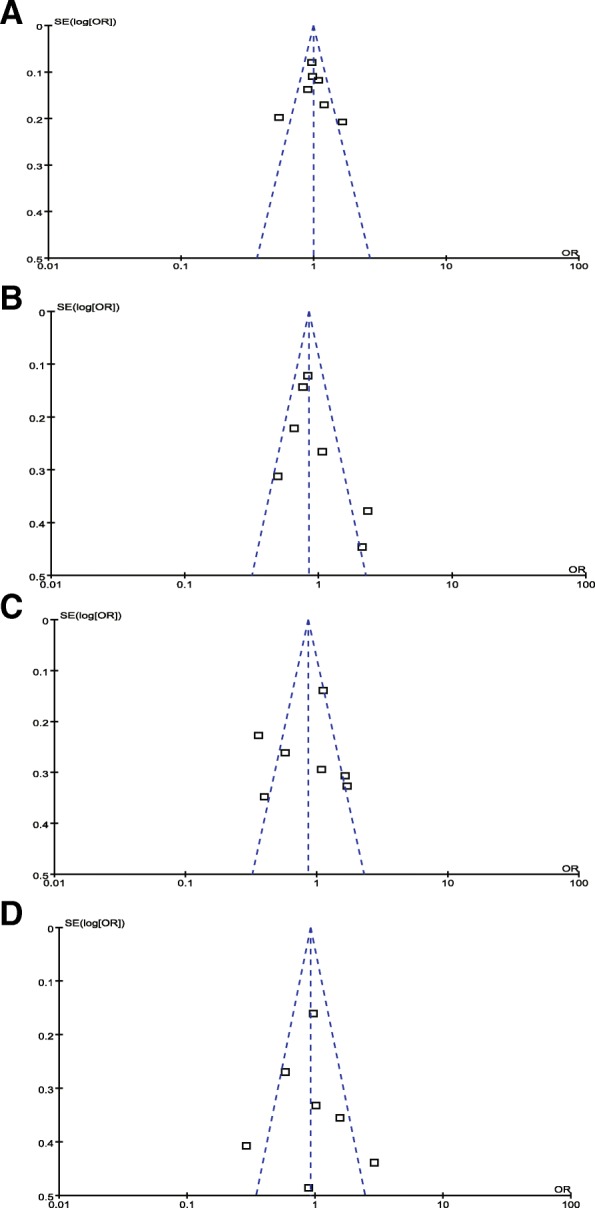
Funnel plot of publication bias for the association between ICAM1 rs5498 and diabetic nephropathy. (**a**): allelic model; (**b**) dominant model; (**c**) recessive model; (**d**) additive model

The Begg’s and Egger’s tests were used to evaluate the publication bias. None of the funnel plots showed any evidence of publication bias (Fig. 4). The *p* value and Z value for Egger’s and Begg’s tests showed no obvious publication bias for ICAM1 rs5498 and DN (Egger’s test: allelic model: *p* = 0.730; dominant model: *p* = 0.346; additive model: *p* = 0.898; recessive model: *p* = 0.738) (Begg’s test: allelic model: *p* = 0.881; dominant model: *p* = 0.293; additive model: *p* = 0.881; recessive model: *p* = 0.881).

## Discussion

The present study demonstrated that the dominant model (GG + AG/AA) of ICAM1 decreased the risk of DN among Caucasian and T1DM subgroups. To the best of our knowledge, this is the first meta-analysis that assessed the genetic association between rs5498 and DN in DM patients. These findings are likely to provide a better and conclusive evaluation of ICAM1 rs5498 for the risk of DN.

Diabetes mellitus (DM) is a heterogeneous deregulation of carbohydrate metabolism, characterized by chronic hyperglycemia that results from impaired glucose metabolism and the subequent increase in blood serum glucose concentration [26, 27]. Multiple factors included metabolic, genetic and inflammatory mediators were shown to be the risk factors for DM [28, 29]. There are three types of DM such as type 1, type 2 and gestational diabetes. Inflammation response has been considered to be a common process both in the pathology of type 1 and type 2 DM [30, 31]. Pro-inflammatory cytokines such as IL-6, IL-18, IL-1 and TNF-α, as well as chemokines including ICAM-1, vascular cell adhesion molecule-1 (VCAM-1) and nuclear transcription factor κB (NFκB) were reported to be significnatly increased in patients with DM [32–34].

Diabetic nephropathy (DN) is one of the microvascular complications in DM and a main cause of end-stage renal disease [35, 36]. DN occurs only in a minority of subjects with either type 1 or type 2 diabetes and seems to result from the interaction between genetic susceptibility and environmental insults [7, 37]. Many studies supporting the contribution of inflammation in diabetes involve immunosuppressive strategies that reduce renal macrophage accumulation and attenuate the development of DN [38, 39]. ICAM-1, a cell-surface protein, which was induced by inflammatory cytokines such as TNF-α, interleukin-1 and interferon-γ. Significantly increased renal expression of ICAM-1 in diabetic rats was deteted during diabetic renal impairment [40]. In addition, an ICAM-1 deficient db/db mouse model of type 2 diabetes showed decreased leukocyte infiltration, reduced glomerular hypertrophy, decreased albuminuria, and decreased tubulo-interstitial fibrosis [13]. Therefore, ICAM1 may play a role in the development of DN.

In the present meta-analysis, we included studies of both T1DM and T2DM patients with and without DN. A significant association was observed between the dominant model of ICAM1 and DN in T1DM, but not in T2DM subgroup, which may indicate that ICAM1 rs5498 was a susceptible factor in T1DM as well as DN. Several groups have reported genetic association studies of ICAM1 rs5498 in T1DM. Guja et al. reported that the transmission of the G allele of SNP K469E (A/G) is increased in Romanian T1DM families [16]. Nishimura et al. showed that this K469E polymorphism is associated with adult-onset T1DM in a Japanese population [26]. However, the association of K469E polymorphism in the ICAM1 gene with T1DM was not found in Danish, Finnish, and British Caucasians [14]. The genetic association between ICAM1 and T1DM-DN was only found in studies conducted by Ma et al. in GoKinD and Swedish Caucasians with 858 cases and 856 controls, which may reduce the power to evaluate the association due to the relatively small sample size. For T2DM-DN, four studies refer to the genetic association between ICAM1 rs5498 and T2DM-DN, while negative result was observed in the present combined analysis.

Notably, significant association was only detected in the Caucasian subgroup, but not in the Asian subgroup. Previous studies indicated that this polymorphism was found to be associated with adult-onset T1DM in Japanese patients [41], but not in Danish, Finnish and British Caucasians [14]. Different genetic backgrounds in Caucasians and Asians might contribute to this inconsistency. Among the included studies, only two studies [17, 18] reported the association between ICAM1 and DN in Caucasian population, which may have affected the real correlation of ICAM1 and DN in Caucasians. Thus, additional studies with larger cohorts are necessary.

Statistically significant heterogeneity across studies was observed in all genotype models (I^2^ ranged from 62 to 95). Significant heterogeneity was also found in Asians, sample size < 1000, and T2D subgroups in all genotype models (I^2^ ranged from 67 to 97). There could be several reasons for these heterogeneities. Firstly, most of these studies had small sample size. Secondly, different strategies were applied in various studies, including control source, population stratification, phenotype selection, genotyping methods, etc.

The limitations of this study should be mentioned. First, the sample size was relatively small, with most studies assessing only a few dozen patients. Large-scale case-controls would have improved the accuracy of the present findings. Secondly, DM types in the patients enrolled in this meta-analysis were somewhat different, since a particular type of DM was not targeted by our inclusion criteria. Thirdly, multiple factors can influence the development of DN, especially the gene-gene interaction and gene-environment interaction, which were not analyzed due to lack of sufficient data. Lastly, we only included studies with two ethnicities. Multiple ethnicity groups need to be assessed in the future to confirm these findings.

## Conclusion

ICAM1 rs5498 may contribute to the susceptibility to DN in dominant model in Caucasians and T1DM patients. More well-designed studies with large sample size and different ethnicities are necessary to confirm this finding.

## Additional file

Additional file 1: **Table S1.** Newcastle-Ottawa scale (NOS) for quality assessment of included studies. (DOC 32 kb)

## Abbreviations

BMI: Body Mass Index
CNKI: Chinese National Knowledge Infrastructure
DBP: Diastolic blood pressure
DM: Diabetes mellitus
DN: diabetic nephropathy
ESRD: end-stage renal disease
HDL: High Density Lipoprotein
HWE: Hardy-Weinberg equilibrium
ICAM1: intercellular adhesion molecule 1
NOS: Newcastle–Ottawa Scale
SBP: Systolic blood pressure
SNP: single nucleotide polymorphism
T1DM: type 1 DM
T2DM: type 2 DM
