# Supplementary material for: Association between E469K polymorphism in the ICAM1 gene and the risk of diabetic nephropathy: a meta-analysis
Source: Lipids Health Dis. 2018 Dec 26;17:293. doi: 10.1186/s12944-018-0922-2 (PMC6307272; doi:10.1186/s12944-018-0922-2)
Supplement: Supplementary file 1 — Table S1. Newcastle-Ottawa scale (NOS) for quality assessment of included studies. (DOC 32 kb) [file 12944_2018_922_MOESM1_ESM.doc]

**Table S1.** Newcastle-Ottawa scale (NOS) for quality assessment of included studies

| Study | Year | Selection | Comparability | Outcome |
| --- | --- | --- | --- | --- |
| Ma | 2008 | **** | ** | *** |
| Ma | 2006 | **** | ** | *** |
| Seman | 2015 | ** | ** | ** |
| Ren | 2015 | **** | * | *** |
| Chen | 2010 | **** | * | *** |
| wang | 2006 | *** | ** | ** |
| Oguz | 2015 | *** | * | ** |
